# Insect odorant receptor trafficking requires calmodulin

**DOI:** 10.1186/s12915-016-0306-x

**Published:** 2016-09-29

**Authors:** Suhyoung Bahk, Walton D. Jones

**Affiliations:** Department of Biological Sciences, Korea Advanced Institute of Science and Technology (KAIST), Daejeon, South Korea

**Keywords:** Insect olfaction, *Drosophila melanogaster*, odorant receptor, Orco, calmodulin

## Abstract

**Background:**

Like most animals, insects rely on their olfactory systems for finding food and mates and in avoiding noxious chemicals and predators. Most insect olfactory neurons express an odorant-specific odorant receptor (OR) along with Orco, the olfactory co-receptor. Orco binds ORs and permits their trafficking to the dendrites of antennal olfactory sensory neurons (OSNs), where together, they are suggested to form heteromeric ligand-gated non-selective cation channels. While most amino acid residues in Orco are well conserved across insect orders, one especially well-conserved region in Orco’s second intracellular loop is a putative calmodulin (CaM) binding site (CBS). In this study, we explore the relationship between Orco and CaM in vivo in the olfactory neurons of *Drosophila melanogaster*.

**Results:**

We first found OSN-specific knock-down of CaM at the onset of OSN development disrupts the spontaneous firing of OSNs and reduces Orco trafficking to the ciliated dendrites of OSNs without affecting their morphology. We then generated a series of Orco CBS mutant proteins and found that none of them rescue the Orco-null *Orco*^*1*^ mutant phenotype, which is characterized by an OR protein trafficking defect that blocks spontaneous and odorant-evoked OSN activity. In contrast to an identically constructed wild-type form of Orco that does rescue the *Orco*^*1*^ phenotype, all the Orco CBS mutants remain stuck in the OSN soma, preventing even the smallest odorant-evoked response. Last, we found CaM’s modulation of OR trafficking is dependent on activity. Knock-down of CaM in all Orco-positive OSNs after OR expression is well established has little effect on olfactory responsiveness alone. When combined with an extended exposure to odorant, however, this late-onset CaM knock-down significantly reduces both olfactory sensitivity and the trafficking of Orco only to the ciliated dendrites of OSNs that respond to the exposed odorant.

**Conclusions:**

In this study, we show CaM regulates OR trafficking and olfactory responses in vivo in *Drosophila* olfactory neurons via a well-conserved binding site on the olfactory co-receptor Orco. As CaM’s modulation of Orco seems to be dependent on activity, we propose a model in which the CaM/Orco interaction allows insect OSNs to maintain appropriate dendritic levels of OR regardless of environmental odorant concentrations.

## Background

Vertebrates and invertebrates alike use their sense of smell to find food and mates and to avoid danger. In *Drosophila*, volatile odorants are detected by odorant receptors (ORs) expressed in olfactory sensory neurons (OSNs) [1, 2]. These OSNs are housed, most often in pairs, within sensory hairs called sensilla that cover the maxillary palps and third antennal segments [3].

Most OSNs express only two ORs—one odorant-specific OR and the olfactory co-receptor Orco [4]. While each of the odorant-specific ORs is expressed in a stereotyped OSN subset [5, 6], Orco seems to be expressed in all OR-expressing OSNs [4]. From its soma, each OSN sends a single dendrite comprising an inner segment, a ciliary constriction, and a branched outer segment into the sensory lymph-filled lumen of an olfactory sensillum [7]. To function, odorant-specific ORs must reach the branched outer dendrites where they can encounter odorant molecules from the environment. Orco is required for OR trafficking past the ciliary constriction into the outer dendrites and, therefore, for normal olfactory responses [4, 8]. Although insect ORs have seven transmembrane domains and appear superficially similar to G protein-coupled receptors (GPCRs), they have a unique inverted membrane topology with cytoplasmic N-termini [8]. Rather than functioning like traditional GPCRs, ORs seem to form odorant-gated non-selective cation channels [9, 10]. Orco physically interacts with the odorant-specific ORs [8] and may also function together with them in olfactory transduction. The various odorant-specific ORs diverge within and across insect species, providing each species with an olfactory repertoire adapted to its particular ecology and odor environment. Orco, in contrast, has been functionally conserved over more than 250 million years of insect evolution, underscoring its central importance in olfaction. Even in species as distantly related as mosquitos and moths, Orco orthologs show up to 70 % identity [11].

One of the best conserved portions of Orco contains a short sequence strongly predicted to bind calmodulin (CaM) [12]. CaM is a well-conserved protein of about 150 amino acids that is ubiquitously expressed in eukaryotes [13, 14]. CaM contains four EF-hand domains that permit it to undergo conformational changes upon binding Ca^2+^ ions. These conformational changes allow CaM to interact with its target proteins, modulating their functions [15–17]. Thus, CaM acts as a sensor that transduces changes in intracellular Ca^2+^ to changes in the function or activity of its target proteins. These target proteins include a wide range of enzymes, cell surface receptors, ion channels, and even structural proteins [18, 19].

Orco’s putative CaM binding site (CBS), coupled with the fact that odorant-evoked activity in olfactory neurons increases intracellular Ca^2+^ [20], made us curious about the physiological relationship between Orco and CaM. Although two recent studies using in vitro and ex vivo preparations with pharmacological inhibitors provided evidence that CaM modulation affects olfactory responses [21, 22], these experiments were limited in their ability to clarify CaM’s in vivo relationship with Orco in insect olfactory neurons as they respond to odorants.

We, therefore, decided to both alter CaM expression at various times during OSN development and generate mutant versions of Orco incapable of binding CaM. We then measured odorant-evoked responses with in vivo extracellular recordings and performed experiments to visualize OSN morphology and OR localization. Here, we show a clear role for CaM in the *Drosophila* olfactory system as a modulator of Orco-mediated OR trafficking to OSN dendrites and, therefore, of olfactory sensitivity. We further show CaM’s modulation of OR trafficking is dependent on OSN activity, meaning OSNs stimulated by odorants show larger defects in olfactory sensitivity upon CaM loss of function. We, therefore, speculate CaM’s sensitivity to intracellular Ca^2+^ levels allows it to enhance Orco-mediated OR trafficking in times of need.

## Results

### OSN-specific CaM knock-down eliminates spontaneous OSN activity

**Fig. 1:**
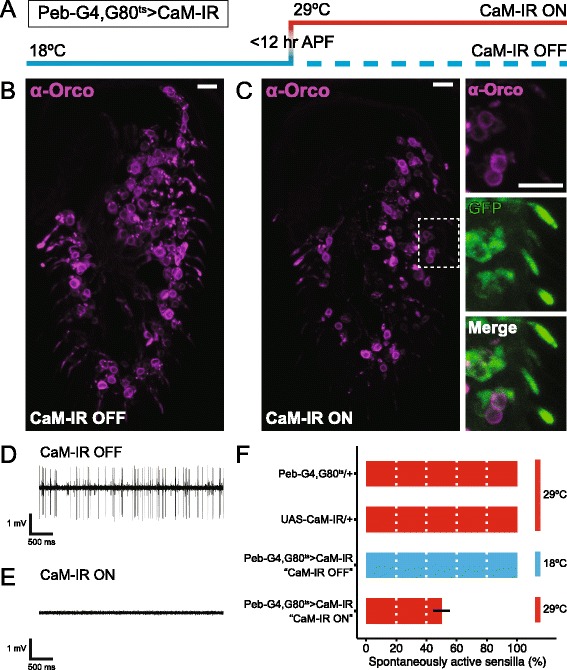
CaM knock-down reduces Orco protein levels and eliminates OSN spontaneous activity. **a** Schematic showing the temperature changes used to restrict CaM knock-down to adult olfactory neurons. At 18 °C, tub-GAL80^ts^ represses *Peb-GAL4*’s activation of *CaM-IR* expression. Since adult OSN development begins 0–12 hours after puparium formation (APF), we chose this time window to shift the *Peb-GAL4, tub-GAL80* ^*ts*^ *> UAS-CaM-IR* flies from 18 °C to 29 °C to inactivate GAL80 and activate CaM knock-down (CaM-IR ON). We maintained the CaM-IR OFF control flies at 18 °C throughout adulthood. **b, c** Staining of antennal sections from *Peb-GAL4, UAS-Dcr-2/+; tub-GAL80* ^*ts*^ */UAS-CaM-IR; UAS-myR::GFP/+* flies with an Orco-specific antibody. *Scale bars*, 10 μm. **b** CaM-IR OFF control flies show normal Orco protein signal in the OSN soma and ciliated dendrites. **c** CaM-IR ON flies show reduced Orco signal in the OSN soma and the ciliated dendrites. This occurs despite normal OSN dendritic morphology, confirmed via visualization of the membrane-tethered myR::GFP in the high-magnification views on the *right*. **d** Sample spike trace from a typical CaM-IR OFF ab2 sensillum. Note the large ab2A spikes and the smaller ab2B spikes. **e** Sample trace from a CaM-IR ON large basiconic sensillum that lacks all spontaneous activity. **f** Percentage of large basiconic sensilla from flies of the indicated genotypes maintained at the indicated temperatures that show spontaneous activity. While all sensilla from the control flies show normal spontaneous activity, only ~50 % of the sensilla from CaM-IR ON flies show spontaneous activity. Data are presented as means ± standard error, *n* = 5 flies, ten sensilla per fly for each genotype. We used the following precise genotypes for this figure: [*Peb-GAL4, UAS-Dcr-2/+ (X); tub-GAL80* ^*ts*^ */+ (II)*], [*UAS-CaM-IR/+ (II)*], and [*Peb-GAL4, UAS-Dcr-2/+ (X); tub-GAL80* ^*ts*^ */CaM-IR (II)*]. *APF* after puparium formation, *CaM* calmodulin, *OSN* olfactory sensory neuron

To explore CaM’s relationship with Orco and olfactory responses in vivo, we first wanted to knock-down CaM in *Drosophila* OSNs as early as possible in their development. To accomplish this, we combined a *UAS-CaM-IR* (inverted repeat) transgene with the peripheral sensory neuron driver *Pebbled-GAL4*. Although it is also expressed in larvae, *Pebbled-GAL4* expression begins in nascent OSNs 12–18 hours after puparium formation (APF) [23], long before the earliest OR expression begins 50–60 hours APF [1]. Combining *Pebbled-GAL4* with *UAS-CaM-IR* (*Peb-G4 > CaM-IR*), however, induces pupal lethality (data not shown). To bypass this lethality, we used the ubiquitously expressed temperature-sensitive *tubulin-GAL80*^*ts*^ to limit expression of the CaM-IR transgene until the pupal stage when OSNs are being born. Thus, we set up and maintained the experimental flies (*Peb-G4, G80*^*ts*^ *> CaM-IR*) and their appropriate controls at 18 °C, where GAL80 suppresses GAL4. Then, to allow GAL4 to drive CaM knock-down, we moved the flies to 29 °C at 0–12 hours APF when OSN development begins [24] (Fig. 1a). We then stained the antennae of these flies with an Orco-specific antibody 10 days post-eclosion. While flies kept at 18 °C (CaM-IR OFF) show normal levels of Orco in both the soma and inner and outer dendrites, flies kept at 29 °C (CaM-IR ON) show reduced Orco staining in many but not all soma and in both the inner and outer ciliated dendrites (Fig. 1b, c). Importantly, this reduction in dendritic Orco occurs despite normal dendritic morphology, confirmed via visualization of a co-expressed membrane-tethered myristoylated green fluorescent protein (GFP) (myR::GFP) (Fig. 1c, magnified images). Note that Pebbled-GAL4 also drives GFP expression in antennal sensory neurons that do not express Orco.

Since Orco is required for the trafficking of ORs to the outer dendrites where they bind odorants, we expected the reduction in Orco trafficking induced by CaM knock-down would also affect OSN activity. We, therefore, used sharpened tungsten electrodes to record the responses of individual large basiconic antennal sensilla. While CaM-IR OFF sensilla show normal spontaneous activity (Fig. 1d), more than 50 % of the CaM-IR ON sensilla are silent, lacking any spontaneous or odorant-evoked action potentials (Fig. 1e, f). This means CaM knock-down prevents Orco from reaching the outer dendrites of the most strongly affected OSNs. Since ab3A neurons that lack their odorant-specific OR (i.e., OR22a) but express Orco retain some spontaneous activity [25], and since Orco is suggested to form functional ion channels that permit a leak current even in the absence of odorant-specific ORs [26], the complete loss of spontaneous activity we see in CaM-depleted OSNs suggests Orco may not even reach the plasma membrane of their soma or inner dendrites. We speculate that this Orco is trapped in the endoplasmic reticulum (ER) because OR22a is retained in Orco-null OSN soma where it co-localizes with an ER marker but not Golgi or lysosome markers [8].

### Orco’s putative CaM binding site

**Fig. 2:**
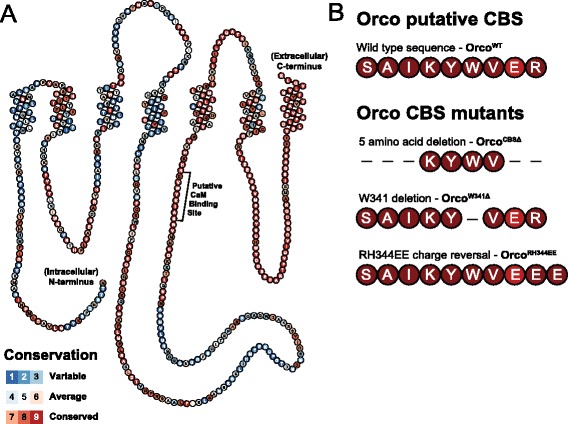
Orco contains a well-conserved putative calmodulin binding site (CBS). **a** Orco snake plot with each amino acid color-coded according to its level of conservation across 78 Orco orthologs. The indicated intracellular CBS falls within one of the most well-conserved regions of Orco. **b** Sequences of the wild-type Orco CBS in Orco^WT^, the N-terminal and C-terminal Orco CBS deletion mutant Orco^CBSΔ^, the single tryptophan deletion mutant Orco^W341Δ^, and the charge reversal mutant Orco^RH344EE^. All the *UAS-Orco* transgenes we generated also have an N-terminal mCherry tag (not shown). *CBS* calmodulin binding site

Next, to confirm the olfactory phenotypes induced by CaM knock-down are a result of Orco malfunction, we generated a series of transgenic fly lines for expressing versions of Orco carrying mutations in its putative CBS. The snake plot in Fig. 2a shows Orco’s predicted transmembrane domains and membrane topology. To produce this plot, we created a multi-protein alignment using Orco orthologs from 78 different insect species and color-coded each amino acid in the consensus sequence based on its relative level of conservation. The second intracellular loop of Orco contains a well-conserved stretch of amino acids that is likely a CBS. This region scores as high as possible in the multifactorial CBS prediction algorithm developed by Yap et al. [12].

Using polymerase chain reaction (PCR) site-directed mutagenesis, we generated four Orco cDNAs containing different versions of the Orco CBS: the wild-type version (Orco^WT^) and three others with mutations of varying severity. These include deletions in the N- and C-termini (Orco^CBSΔ^), a single amino acid deletion (Orco^W431Δ^), and charge reversals of two positive residues (Orco^RH344EE^) (Fig. 2b). We cloned each of these Orco variants into an upstream activating sequence (UAS) transgenesis vector modified to add an N-terminal mCherry tag to each resulting Orco protein. Then, using standard fly transgenesis techniques and site-specific recombination, we inserted each vector into the identical location on the second chromosome to match expression levels between variants.

### Orco CBS mutations disrupt OR trafficking

**Fig. 3:**
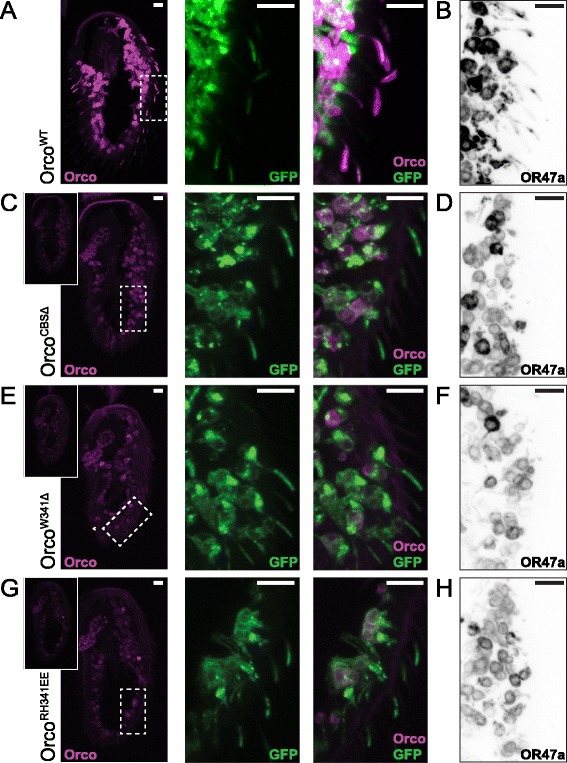
Mutation of the Orco CBS disrupts Orco and OR47a trafficking to the ciliated OSN dendrites. **a** *UAS-mCherry::Orco* ^*WT*^ */UAS-myR::GFP (II); Orco-GAL4, Orco* ^*1*^ *(III)* flies show normal Orco protein localization in OSN soma and ciliated dendrites (*magenta*) and normal dendritic morphology (*green*). We confirmed this by visualization of mCherry and GFP, respectively. **b** Staining of *UAS-mCherry::Orco* ^*WT*^ */UAS-EGFP::OR47a (II); Orco-GAL4, Orco* ^*1*^ *(III)* antennae with an anti-GFP antibody shows normal trafficking of OR47a protein to the ciliated outer dendrites. **c–h** We then examined the localization of Orco and OR47a proteins in flies expressing Orco^CBSΔ^ (**c, d**), Orco^W341Δ^ (**e, f**), and Orco^RH344EE^ (**g, h**). For unknown reasons, perhaps increased protein degradation, the Orco CBS mutants all show lower mCherry signal than Orco^WT^ when imaged at the same confocal settings (**c, e, g** *left inset panels*). After boosting the confocal gain, it was clear none of the three Orco CBS mutants rescue the localization of Orco to the outer dendrites (**c, e, g** *magenta*) despite normal dendritic morphology (**c, e, g** *green*). Besides failing to rescue the localization of OR47a protein to the ciliated outer dendrites, expression of the Orco CBS mutants also seems to reduce OR47a staining in the OSN soma (**d, f, h**). For **b**, **d**, **f**, and **h**, the color of each image is inverted to improve dendrite visibility. All *scale bars*, 10 μm. *CBS* calmodulin binding site, *GFP* green fluorescent protein, *OSN* olfactory sensory neuron

Next, we used *Orco-GAL4* to drive OSN-specific expression of each *UAS-Orco* transgene in the *Orco*^*1*^ null-mutant background. As expected, by visualizing its N-terminal mCherry tag, we found Orco^WT^ protein is expressed well and localized normally to both the OSN soma and ciliated dendrites (Fig. 3a). A membrane-tethered GFP also revealed normal dendritic morphology (Fig. 3a). When we attempted to visualize the N-terminal mCherry tags of the three Orco CBS mutant proteins at the same confocal settings we used for Orco^WT^, we observed much lower signal levels in the OSN soma (Fig. 3c, e, g, insets). By boosting the signal gain, it became clear that all three Orco CBS mutants remain stuck in the OSN soma, unable to traffic to the ciliated dendrites (Fig. 3c, e, g). With all three mutants, however, we confirmed proper dendritic localization of a membrane-tethered GFP, which shows the defect in Orco localization is not due to changes in OSN morphology (Fig. 3c, e, g). Since we cloned all four Orco cDNA variants in the same way and inserted them into the same genomic location, we suspect the reduced signal from the Orco CBS mutant proteins results from increased protein degradation rather than reduced transcription.

Because Orco is necessary for the dendritic localization of odorant-specific ORs, we next asked whether the Orco CBS mutants can support OR localization. To address this, we again used *Orco-GAL4* to drive OSN-specific expression of each version of Orco along with enhanced green fluorescent protein (EGFP)-tagged OR47a in the *Orco*^*1*^ null-mutant background. While OR47a protein localization is normal in flies expressing Orco^WT^ (Fig. 3b), it remains stuck in the soma of OSNs expressing any of the three Orco CBS mutants (Fig. 3d, f, h). Interestingly, OSNs expressing the Orco CBS mutant proteins also show slightly less OR47a protein in their soma than OSN expressing Orco^WT^. This is reminiscent of the reduced OR22a protein staining observed in Orco-null OSNs [4] and suggests Orco’s interaction with the ORs is required to maintain OR protein stability. For the images in Fig. 3d, f, and h, we used an antibody to stain the EGFP tag on the OR47a protein, inverting the colors to improve the visibility of the thin ciliated outer dendrites. Since none of the Orco CBS mutants support OR localization to the ciliated outer dendrites, it is unsurprising that OSNs expressing them are just as electrophysiologically silent as *Orco*^*1*^ mutant OSNs (data not shown).

**Fig. 4:**
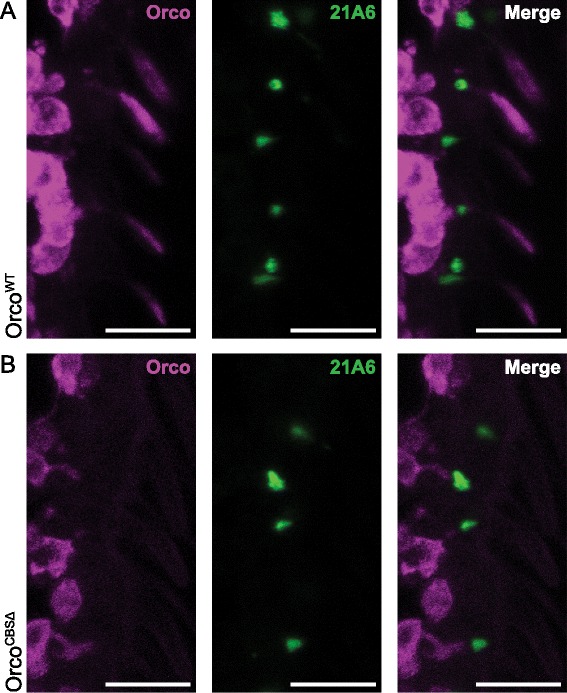
CBS mutant Orco cannot enter the ciliated outer dendrites. **a** Staining of *UAS-mCherry::Orco* ^*WT*^ *(II); Orco-GAL4, Orco* ^*1*^ *(III)* antennae with the 21A6 antibody, a marker of the ciliary base, reveals normal Orco trafficking from the OSN soma all the way to the ciliated outer dendrites. **b** Orco trafficking in *UAS-mCherry::Orco* ^*CBSΔ*^ *(II); Orco-GAL4, Orco* ^*1*^ *(III)* flies is blocked at the ciliary base. Some Orco is visible in the OSN soma and inner dendritic segments, but nothing passes the ciliary constriction marked by the 21A6 signal. In **a** and **b**, we visualized Orco^WT^ and Orco^CBSΔ^ proteins using their mCherry tags, but we used a higher gain on the confocal microscope for Orco^CBSΔ^. All *scale bars*, 10 μm. *OSN* olfactory sensory neuron

We next stained antennae from flies carrying *Orco-GAL4* and either *UAS-Orco*^*WT*^ or *UAS-Orco*^*CBSΔ*^ with an antibody that marks the base of each cilium (i.e., 21A6). We found that Orco^WT^ protein extends from the soma to the inner dendritic segment and the outer ciliated dendritic segment (Fig. 4a). In contrast, Orco^CBSΔ^ protein is visible only in the soma and inner dendritic segment, but absent from the outer ciliated dendritic segment (Fig. 4b). Thus, Orco CBS mutations prevent Orco from passing the base of each olfactory cilium, trapping it and its OR partners in the soma and inner dendritic segments where they cannot encounter odorants.

It is possible mutations in the Orco CBS disrupt the three-dimensional structure of Orco, meaning the defect in OR trafficking we observed is due to a lack of functional Orco rather than a specific disruption of Orco’s ability to bind CaM. This is unlikely, however, because all three Orco CBS mutants—including the single amino acid deletion Orco^W341Δ^—produce the same phenotype. We also found all three Orco CBS mutant transgenes produce proteins in vivo with structures normal enough that they can be recognized by Orco-specific antibodies (data not shown). In addition, heterologous cells expressing a similar Orco mutant with a single amino acid substitution in its CBS (Orco^K339N^) respond to a non-odorant Orco agonist, albeit more weakly than cells expressing wild-type Orco [21]. Also, the similarity between our results with these Orco CBS mutants and the CaM depletion-induced disruption of Orco trafficking and OSN spontaneous activity strongly supports a role for CaM in the modulation of Orco/OR trafficking to the outer ciliated dendrites of OSNs.

### Prolonged odorant exposure enhances the effect of CaM knock-down

We were curious how this role in Orco/OR trafficking could fit with CaM’s role as a calcium sensor, especially considering how odorant exposure induces action potentials and action potentials increase intracellular calcium. It seems counterintuitive that odorant activation could enhance OR trafficking given the phenomena of sensory adaptation and behavioral habituation. Sensory adaptation occurs within OSNs, rapidly reducing their firing frequencies upon continuous or repeated odorant stimulation and reducing the magnitude of subsequent electrophysiological responses to the same or similar odorants. This adaptation lasts on the order of seconds to minutes [27] and is specific to individual OSN subtypes and the ORs they express [28]. Unfortunately, the molecular mechanisms of sensory adaptation in *Drosophila* are not yet well understood, but they likely involve OR post-translational modification, degradation, or internalization, as in the mammalian olfactory system [29]. Olfactory habituation, in contrast, is characterized by a long-lasting reduction in odorant-evoked *behavioral* responses following long-term exposure to high concentrations of identical or similar odorants. Habituation occurs via changes in the activity of lateral inhibitory interneurons in the antennal lobes that alter the projection neuron output into higher brain centers, rather than via changes in the OSNs themselves [30, 31]. Interestingly, although flies exposed to a high concentration (10^-1^ v/v) of benzaldehyde for 4 days show strong behavioral habituation for several days post-exposure, their peripheral electrophysiological responses to benzaldehyde are normal only 1 day post-exposure [32]. Thus, upon continuous odorant stimulation, OSNs undergo peripheral sensory adaptation and, as the odorant exposure is prolonged, it induces central habituation. During or after a long-term odorant exposure, however, peripheral sensitivity returns to normal, despite the flies continuing to show reduced behavioral responses. We, therefore, wondered if CaM may contribute to maintaining olfactory sensitivity in the antenna upon prolonged odorant exposure by maintaining adequate levels of OR in the ciliated OSN dendrites in an activity-dependent manner. This would be analogous to CaM’s neuronal activity-dependent regulation of the CaM-dependent protein kinases (CaMKs) [33] and to CaM’s regulation of both mGluR5 and EGFR trafficking [34, 35].

**Fig. 5:**
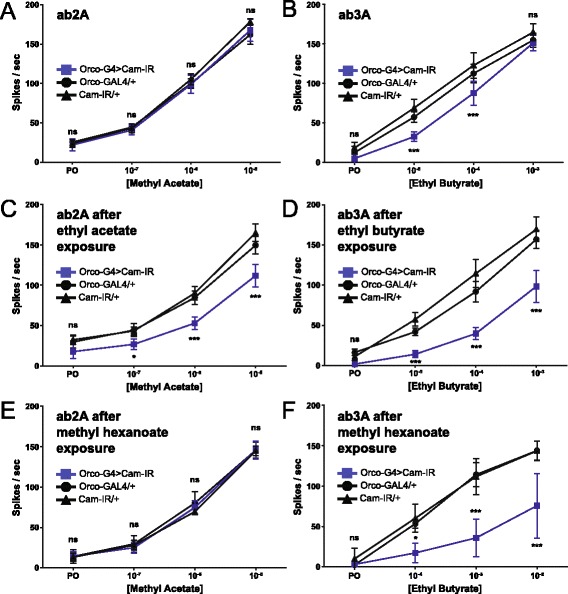
CaM knock-down induces an activity-dependent reduction in olfactory sensitivity. **a, b** CaM knock-down after OR expression has begun using *Orco-GAL4* does not affect the sensitivity of ab2A neurons to methyl acetate (**a**) and slightly reduces the sensitivity of ab3A neurons to ethyl butyrate (**b**). **c** When we maintained the same flies in vials containing 10 % ethyl acetate, which activates ab2A, we found CaM knock-down produces a dramatic reduction in the sensitivity of ab2A neurons to methyl acetate. **d** When we maintained these flies in vials containing 10 % ethyl butyrate, we observed a similar but even more significant reduction in the sensitivity of ab3A to ethyl butyrate. **e, f** This effect of CaM-IR is OSN subtype-specific, as long-term exposure to 10 % methyl hexanoate—a ligand for ab3A but not ab2A—does not affect ab2A sensitivity (**e**) but strongly reduces ab3A sensitivity (**f**). For all graphs, data are presented as means ±95 % confidence intervals. *n* ≥ 8 sensilla for each genotype and odorant dose. Asterisks indicate significant differences between the experimental line and both controls, with the higher of the two *P* values determining the number of asterisks (two-way ANOVA; Bonferroni post-hoc test; * *P* < 0.05, ** *P* < 0.01, and *** *P* < 0.001). We used the following precise genotypes: [*UAS-Dcr-2/UAS-CaM-IR (II); Orco-GAL4 (III)*], [*UAS-Dcr-2/+ (II); Orco-GAL4 (III)*], and [*UAS-CaM-IR/+ (II)*]. *CaM* calmodulin, *ns* non-significant, *OSN* olfactory sensory neuron, *OR* odorant receptor

Figure 1 shows how *Peb-GAL4*-driven CaM knock-down, beginning 12–18 hours APF, long before Orco is expressed, can eliminate OSN spontaneous activity. *Orco-GAL4* expression begins around 80 hours APF when Orco expression begins [4]. When we used *Orco-GAL4* to knock down CaM, we observed no change in the electrophysiological dose–response curves for ab2A neurons responding to methyl acetate (Fig. 5a) and only a small drop in the lower dose–responses for ab3A neurons responding to ethyl butyrate (EB) (Fig. 5b). In contrast, ab2A neurons in CaM-depleted flies exposed to 7 days of 10 % ethyl acetate, which activates ab2A, show a strong reduction in methyl acetate sensitivity at all doses (Fig. 5c). Here, we used ethyl acetate instead of methyl acetate because methyl acetate evaporates rapidly, making long-term exposures difficult. Similarly, ab3A neurons in CaM-depleted flies exposed to 7 days of 10 % EB show a dramatic drop in sensitivity across all doses tested (Fig. 5d). To determine whether this effect is OSN-specific, we exposed flies to 10 % methyl hexanoate, which activates ab3A but not ab2A, and measured the sensitivity of ab2A neurons to methyl acetate and ab3A neurons to EB. As expected, methyl hexanoate exposure affects only the sensitivity of the CaM-depleted ab3A neurons (Fig. 5e, f).

### Prolonged odorant exposure reduces Orco in the outer dendrites

**Fig. 6:**
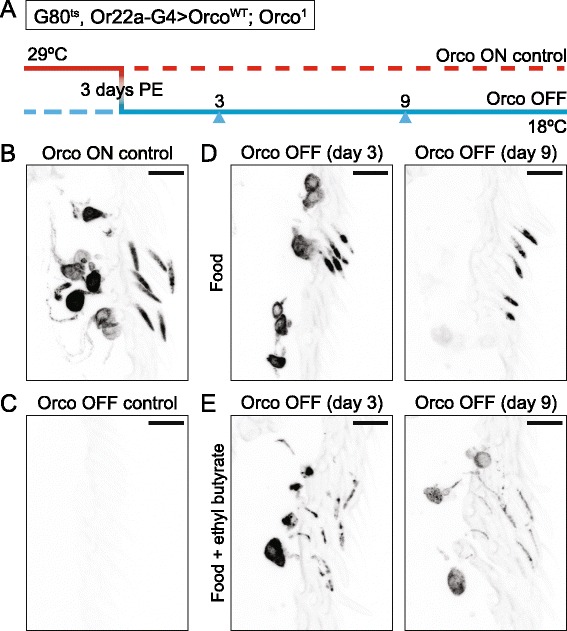
Odorant exposure in the absence of new Orco production accelerates dendritic Orco depletion. **a** Experimental scheme. We maintained *UAS-mCherry::Orco* ^*WT*^ */tub-GAL80* ^*ts*^ *, Or22a-GAL4 (II); tub-GAL80* ^*ts*^ *, Orco* ^*1*^ *(III)* flies at 29 °C until 3 days post-eclosion. Then, after transferring them to 18 °C to block additional Orco expression, we divided the flies into two groups: those maintained on normal food and those maintained on food plus a perforated microcentrifuge tube containing 10 % ethyl butyrate. **b**–**e** Orco protein localization in ab3A dendrites visualized via its mCherry tag. **b** Orco ON control antennae prior to the transfer to 18 °C show clear ab3A soma and outer dendritic localization of Orco. **c** Orco OFF control antennae from flies maintained continuously at 18 °C show no Orco expression, indicating three copies of *tub-GAL80* ^*ts*^ suffice to block Orco expression completely at the restrictive temperature. **d** When new Orco production is blocked, existing Orco seems to move gradually from the ab3A soma to the outer dendrites. Three days after the transfer to 18 °C, the ab3A soma shows slightly less Orco and the outer dendrites slightly more (*left*). By day 9, the ab3A soma is empty while the outer dendrites show only slightly lower levels of Orco (*right*). **e** When new Orco production is similarly blocked in the presence of the ab3A ligand ethyl butyrate, the level of outer dendritic Orco is reduced even after only 3 days. This is accompanied by enhanced signal in ab3A soma (*left*). By day 9, the ab3A outer dendrites are nearly depleted of Orco, but residual Orco is still readily visible in the OSN soma. For **b**–**e**, the color of each image is inverted to improve outer dendrite visibility. All *scale bars*, 10 μm. *PE* post-eclosion

To explore the mechanism by which odorant exposure enhances the CaM knock-down phenotype, we needed to know whether prolonged odorant exposure alone can reduce the level of Orco in the ciliated OSN dendrites. We, therefore, decided to modify an experiment Benton et al. performed to show Orco is required in adult OSNs to maintain OR localization in the ciliated dendrites [8]. In this experiment, we used *Or22a-GAL4* to drive expression of Orco^WT^, rescuing the *Orco*^*1*^ mutation only in ab3A neurons. These flies also carried three copies of the temperature-sensitive *tub-GAL80*^*ts*^, allowing us to block any new Orco expression by shifting the flies from 29 °C to 18 °C (Fig. 6a). In the antennae of Orco ON control flies raised continuously at 29 °C, we observed a strong mCherry::Orco signal in both the soma and outer ciliated dendrites of their ab3A neurons (Fig. 6b). In contrast, the Orco OFF control, raised continuously at 18 °C, showed absolutely no Orco signal at all (Fig. 6c), confirming GAL80’s function. To see how odorant exposure affects the level of Orco protein in the outer dendrites, we reared these flies at 29 °C to allow Orco expression. Three days post-eclosion, we moved the flies to either vials containing normal food or normal food plus a small perforated tube containing 10 % EB, which activates ab3A neurons. We then moved both sets of vials to 18 °C to block additional Orco expression, visualizing Orco in both the soma and outer ciliated dendrites 3 or 9 days later (Fig. 6a). After 3 days in the absence of EB, the level of Orco protein is slightly reduced in ab3A OSN soma and increased in the outer ciliated dendrites, presumably because Orco is being continuously shuttled there. After 9 days in the absence of EB, while the ab3A soma lacks any Orco protein, some residual Orco remains in the ciliated dendrites (Fig. 6d). Prolonged exposure to EB dramatically changes this picture. After 3 days of EB exposure, there is more Orco protein in the ab3A soma and less in the outer dendrites. After 9 days of EB exposure, the ab3A soma still shows significant Orco protein, but the outer dendrites are nearly empty (Fig. 6e). Thus, extended odorant exposure seems to induce internalization and perhaps degradation of Orco, depleting the outer ciliated dendrites.

### Odorant exposure enhances Orco loss in CaM-depleted OSNs

**Fig. 7:**
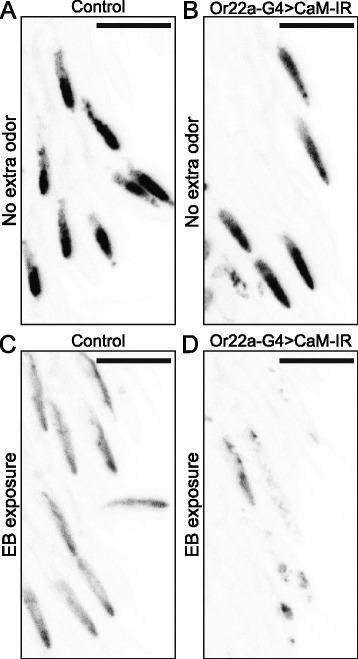
CaM knock-down reduces dendritic Orco in an activity-dependent manner. **a** The ab3A outer dendrites of [*UAS-Dcr-2/+ (X); UAS-mCherry::Orco* ^*WT*^ */+ (II); Or22a-GAL4, Orco* ^*1*^ *(III)*] control flies show normal Orco localization, as determined by visualizing mCherry. **b** The ab3A outer dendrites of [*UAS-Dcr-2/+ (X); UAS-mCherry::Orco* ^*WT*^ */UAS-CaM-IR (II); Or22a-GAL4, Orco* ^*1*^ *(III)*] flies show only a slight reduction in Orco when they are maintained for 7 days on normal food. **c, d** Identical flies as in (**a**) and (**b**) maintained for 7 days in food vials with a perforated tube containing 10 % ethyl butyrate. **c** Long-term odorant exposure significantly reduces outer dendritic Orco in control flies. **d** Combining long-term odorant exposure with CaM knock-down nearly eliminates Orco in the outer dendrites of ab3A OSNs. The color of all images is inverted to improve outer dendrite visibility. All *scale bars*, 10 μm. *CaM* calmodulin, *EB* ethyl butyrate

Next, we wanted to confirm that the reduced responsiveness of CaM-depleted OSNs after a long-term odorant exposure is accompanied by reduced localization of Orco to the ciliated outer dendrites. To do so, we used *Or22a-GAL4* to drive both Orco^WT^ and *CaM-IR* in the *Orco*^*1*^ null mutant background and compared the level of Orco protein in the ab3A outer dendrites to that of controls lacking *CaM-IR*. Without long-term EB exposure, the ab3A outer dendrites of control flies (Fig. 7a) show Orco levels similar but slightly higher than the ab3A outer dendrites of *Or22a-G4 > CaM-IR* flies (Fig. 7b). After long-term exposure to 10 % EB, however, the ab3A outer dendrites of control flies (Fig. 7c) contain much more Orco than the ab3A outer dendrites of *Or22a-G4 > CaM-IR* flies (Fig. 7d). This is consistent with the electrophysiological results shown in Fig. 5. Together, these results suggest CaM’s modulation of Orco trafficking depends on OSN activity levels, and that its role becomes especially important during long-term odorant exposures that deplete the dendrites of specific OSN subtypes of their supply of Orco and hence active Orco/OR complexes.

## Discussion

Although two previous studies provided evidence that CaM modulates Orco activation and olfactory sensitization, they primarily relied on a non-odorant Orco agonist and pharmacological CaM inhibition in in vitro and ex vivo experiments [21, 22]. Although still distinct, these functions for CaM are somewhat reminiscent of CaM’s role in vertebrate olfaction where it modulates the signaling pathways downstream of OR activation. Since vertebrate ORs are typical GPCRs, odorant binding activates a second messenger cascade that produces cAMP and activates cyclic nucleotide-gated (CNG) channels to alter neuronal activity. Not only does CaM directly modulate the CNG channels and, therefore, the activity of vertebrate olfactory neurons [36], it indirectly modulates the olfactory adenylate cyclase via CaMKII, affecting olfactory adaptation [37]. Here, in contrast, we uncovered a previously unknown role for CaM in concert with the olfactory co-receptor Orco in trafficking insect ORs to the ciliated dendrites of olfactory sensory neurons in vivo. We showed CaM knock-down in OSNs prior to their expression of ORs dramatically disrupts the trafficking of Orco, interfering with OSN activity (Fig. 1). We also showed mutations in the putative Orco CBS completely block Orco and odorant-specific OR trafficking (Figs. 2 and 3). We were able to narrow the location of this block to the ciliary constriction that divides the inner dendritic segment from the ciliated outer dendrites (Fig. 4).

As a calcium sensor, CaM likely responds to the increases in intracellular calcium that occur with odorant-evoked OSN activity. Thus, after establishing a role for CaM in OR trafficking, we wondered whether CaM may be important in maintaining peripheral responsiveness to odorants during long-term odorant exposure. Indeed, we found long-term odorant exposure enhances the reduction in sensitivity induced by CaM knock-down in an odorant- and OSN subtype-specific manner (Fig. 5). We then tried to clarify the mechanism of this loss of sensitivity induced by loss of CaM. We wondered if long-term odorant exposure may reduce the level of Orco in the ciliated dendrites and, hence, increase the demand for CaM-induced Orco trafficking. Indeed, we found this to be the case; long-term odorant exposure in the presence of CaM but in the absence of new Orco production seems to induce the movement of Orco from the ciliated dendrites back into the soma, presumably by inducing receptor internalization and subsequent recycling or degradation (Fig. 6). In CaM-depleted OSNs, the activity-dependent reduction in sensitivity is accompanied by a dramatic reduction in outer dendritic Orco localization (Fig. 7).

Thus, our results demonstrate a role for CaM in trafficking Orco/OR complexes to the outer ciliated dendrites of OSNs. This role for CaM is especially important when OSNs are subjected to prolonged odorant exposure, because odorant exposure seems to deplete Orco in the outer dendrites, making it necessary to send out additional Orco/OR complexes to maintain olfactory sensitivity. Our results suggest a model whereby OSNs can maintain optimal sensitivity despite two opposing forces continuously altering the abundance of ORs in the outer dendrites. Strong OSN activation reduces Orco/OR levels in the outer dendrites, but the resulting increase in intracellular Ca^2+^ and subsequent CaM activation induces further outward trafficking of Orco and ORs, making up for the loss.

Such an activity-dependent regulation of Orco trafficking during and after prolonged odorant exposure seems more reasonable upon consideration of a fly’s olfactory environment. A fly searching for food first encounters low concentrations of fruity esters emanating from a piece of rotting fruit. The fly must then move through plumes of these odorants, tracking their concentration gradients to their source. This would involve repeated transient exposures to low but gradually increasing concentrations of odorants. Once the fly lands on a food source, however, it is immersed in much higher concentrations of odorants that induce sensory adaptation and even central habituation. These odorant-specific reductions of both peripheral and central responsiveness are presumably important in allowing the fly to shift attention to other important olfactory stimuli, like potential mates or danger signals. Peripheral sensitivity should not remain too low for too long, however, because this would prevent the fly from finding the same food source again if it were forced to leave. Thus, CaM’s activity dependence seems to ensure a steady flow of OR protein into the outer dendrites of OSNs that are being consistently activated. It will be interesting to see whether CaM represents a key hub for the context-dependent modulation of olfactory sensitivity. For example, activation of CaM-induced Orco/OR trafficking even in the absence of prolonged odorant exposure may increase olfactory sensitivity to food-related odorants when a fly is hungry, while its inhibition could reduce sensitivity to sex pheromones after mating.

Despite uncovering a new role for CaM in Orco trafficking, many unanswered questions remain. We are curious, for example, whether proper dendritic trafficking of ORs requires signaling pathways and proteins other than CaM and Orco. How do CaM and Orco work together with the highly conserved multi-protein IFT complexes that comprise the ciliary trafficking machinery? How does CaM physically interact with Orco? Does it bind continuously or does it simply help deliver Orco to a specific subcellular location? Does CaM enter the outer dendrites itself, and if so, does it have roles in Orco modulation in vivo apart from its effect on trafficking? Answers to these and more questions await further experiments, but attaining some of these answers will likely require novel heterologous systems that more closely resemble the highly specialized microenvironment of insect OSNs. We hope our contribution to a clearer understanding of OR trafficking will help push us forward toward this goal. It will also be interesting to see whether the ciliary trafficking of other important sensory receptors is similarly modulated by CaM.

## Conclusions

Insects, both the beneficial and harmful, depend on their olfactory systems as they search for food and mates and as they avoid danger. Insect olfaction depends on the proper trafficking of Orco/OR complexes to the ciliated OSN dendrites where they encounter odorants. Using a variety of genetic tools, we discovered a previously unknown role for the calcium sensor CaM as a modulator of Orco/OR trafficking. Both knock-down of CaM and mutation of Orco’s CBS disrupt the localization of Orco to the outer ciliated dendrites of OSNs. This affects the spontaneous activity of OSNs and their responsiveness to odorants. CaM’s modulation of Orco is also dependent on OSN activity, in that prolonged odorant exposure depletes Orco from the ciliated dendrites and magnifies the importance of CaM-dependent Orco trafficking in maintaining olfactory sensitivity. We expect our current study and future in vivo studies like it will improve our understanding of the unique microenvironment of insect OSNs and someday make it possible to manipulate insect olfaction in ways beneficial to humanity.

## Methods

### Fly stocks

We maintained our fly stocks at either 18 °C or 25 °C and 60 % humidity under a 12 h:12 h light:dark cycle. We used standard cornmeal-yeast-corn syrup medium (http://flystocks.bio.indiana.edu/Fly_Work/media-recipes/bloomfood.htm) containing 1.5 g Tegosept per liter of food as an anti-fungal agent.

We received the *Orco-GAL4* and *Pebbled-GAL4* strains as generous gifts from Bill Hansson (Jena, Germany) and Mattias Alenius (Linköping, Sweden), respectively. We obtained the following fly strains from the Bloomington Stock Center (Bloomington stock numbers given in parentheses): *Orco*^*1*^ (23129), *10XUAS-myR::GFP* (32198), *tub-GAL80*^*ts*^ (7019), *tub-GAL80*^*ts*^ (7018), *UAS-Dcr2* (24648), *UAS-Dcr2* (24650), and *Or22a-GAL4* (9952). We obtained the following fly stock from the VDRC Stock Center: *UAS-CaM-IR* (stock number: 102004).

To generate the Orco CBS mutants, we designed primers for mutagenizing a pGEMT-easy vector containing Orco cDNA. These primers, presented 5′ to 3′, were: *UAS-Orco*^*CBSΔ*^ (GATGATGGTGCGCAAGTACTGGGTC and GACCCAGTACTTGCGCACCATCATC for the N-terminal "S-A-I" deletion, CAAGTACTGGGTCCACAAGCACGTG and CACGTGCTTGTGGACCCAGTACTTG for C-terminal "E-R" deletion), *UAS-Orco*^*W341Δ*^ (GTGCCATCAAGTACGTCGAGCGGCACAAG and CTTGTGCCGCTCGACGTACTTGATGGCAC), and *UAS-Orco*^*RH344EE*^ (CATCAAGTACTGGGTCGAGGAGGAGAAGCACGTGGTGCGACTG and CAGTCGCACCACGTGCTTCTCCTCCTCGACCCAGTACTTGATG). After confirming their sequences, we subcloned each mutated cDNA into a modified attB-containing SST13 UAS vector [38] downstream of and in-frame with the coding sequence for mCherry. We also subcloned a wild-type Orco cDNA into this same vector to produce the *UAS-Orco*^*WT*^ control. We performed this subcloning into the modified SST13 UAS vector using the sequence and ligation-independent cloning (SLIC) protocol [39, 40] and the following primers presented 5′ to 3′: GCGGCCGCGGCTCGAGAAACAACCTCGATGCAGCCGA and ATATGGTACCCTCGAGTTACTTGAGCTGCACCAGC.

### The Orco snake plot

We used Geneious [41] to align Orco orthologs from 78 different insect species available from the National Center for Biotechnology Information. We then used Consurf [42] to transform this multiple sequence alignment into conservation scores for each individual amino acid of the Orco consensus sequence. Using transmembrane prediction data produced by the TMHMM algorithm [43], we created a snake plot for *Drosophila melanogaster* Orco using the protein visualization tool Viseur (Nancy, France). Finally, we color-coded each amino acid according to its conservation score using Adobe Illustrator.

### Single-sensillum electrophysiology

We performed single-sensillum recordings from *Drosophila* ab2 and ab3 sensilla as previously described [44]. We used paraffin oil (76235, Sigma-Aldrich) for serial dilutions of methyl acetate (45999, Sigma-Aldrich) and ethyl butyrate (E15701, Sigma-Aldrich). We chose these odorants for their selective activation of the ab2A and ab3A neurons [45].

### Immunostaining of antennal sections

We cut 14 μm frozen sections from fly antennae and prepared and stained them as previously described [4]. We used the following antibodies: mouse anti-GFP (A11120, ThermoFisher Scientific) at 1:200, Alexa 488-conjugated anti-mouse IgG (ab150117, Abcam) at 1:850, Alexa 594-conjugated anti-rabbit IgG (A11012, ThermoFisher Scientific) at 1:850, the mouse monoclonal 21A6 (DSHB) at 1:100, and a polyclonal rabbit anti-Orco antibody (raised against the peptide SSIPVEIPRLPIKS by AbFrontier, South Korea) at 1:3000.

### Odorant exposure protocol

After aging each group of flies on normal food for 3 days post-eclosion, we moved them to new vials containing an odorant source we exchanged daily. Each odorant source comprised a perforated PCR tube containing 200 μL of odorant diluted 1:10 (10^-1^ v/v) in paraffin oil placed inside a larger perforated microcentrifuge tube. All the odorants for this experiment were from Sigma-Aldrich. These included ethyl acetate (34858), ethyl butyrate (E15701), and methyl hexanoate (259942).

### Statistical analysis

To identify statistically significant differences in olfactory dose–response curves, we compared the experimental genotype to its controls at each odorant concentration using two-way ANOVAs with Bonferroni post-hoc tests for multiple comparisons. For each odorant concentration, the higher of two *P* values is indicated on the graph. We indicated statistically significant results with asterisks (* *P* ≤ 0.05, ** *P* ≤ 0.01, and *** *P* ≤ 0.001) and non-significant results with *ns*. We performed all data analysis in either Graphpad (San Diego, CA, USA) or using R [46].

## Abbreviations

APF: After puparium formation
CaM: Calmodulin
CaMK: CaM-dependent protein kinase
CBS: Calmodulin binding site
EB: Ethyl butyrate
GFP: Green fluorescent protein
OR: Odorant receptor
OSN: Olfactory sensory neuron.
